# miR‐448‐3p/miR‐1264‐3p Participates in Intermittent Hypoxic Response in Hippocampus by Regulating Fam76b/hnRNPA2B1

**DOI:** 10.1111/cns.70239

**Published:** 2025-02-06

**Authors:** Chuncheng Liu, Donghui Qu, Chaoxun Li, Wenhua Pu, Jun Li, Lu Cai

**Affiliations:** ^1^ School of Life Science and Technology Inner Mongolia University of Science & Technology Baotou China; ^2^ Inner Mongolia Key Laboratory of Life Health and Bioinformatics Baotou China

**Keywords:** alternative splicing, Fam76b, hnRNPA2B1, intermittent hypoxia, miRNA

## Abstract

**Background:**

Intermittent hypoxia (IH), as a key pathogenic factor of obstructive sleep apnea syndrome (OSAS), can cause many diseases, such as increased inflammation and oxidative stress, diabetes, cardiovascular disease, and Alzheimer's disease (AD). The response of cells to hypoxia involves multiple levels of regulatory mechanisms, including transcriptional regulation of gene expression, regulation of mRNA stability, post‐transcriptional regulation, and post‐translational modification regulation.

**Aims:**

The regulation of miRNA and alternative splicing (AS) in neuronal response to intermittent hypoxia deserve further study.

**Materials & Methods:**

By establishing a mouse model of intermittent hypoxia, we conducted functional studies on key miRNAs and splicing factor using methods such as miRNA sequencing, bioinformatics, and molecular biology.

**Results:**

In the mouse hippocampus, intermittent hypoxia altered the expression of many miRNAs, with miR‐448‐3p and miR‐1264‐3p changing over the course of more than three time periods. Interestingly, the expression of *Fam76b*, the common target gene of these two miRNAs, also changed under intermittent hypoxia. Further studies showed that Fam76b may regulate the ratio of *Nbr1* and *Dph3* transcripts in response to hypoxia by affecting the localization of hnRNPA2B1 protein within cells.

**Discussion:**

Research into intermittent hypoxia‐induced disorders, including Alzheimer's disease and other neurodegenerative diseases, might benefit from a better understanding of the regulatory mechanisms of miRNA and alternative splicing in hypoxic response at the animal and cell levels.

**Conclusion:**

This study demonstrates that intermittent hypoxia alters the expression of miR‐448‐3p and miR‐1264‐3p, as well as the localization of the splicing factor hnRNPA2B1 in the cell nucleus. These findings enhance our understanding of the molecular mechanisms of neuronal responses to hypoxia and hold potential implications for treating hypoxia‐related diseases like Alzheimer's disease.

## Graphical abstract

In the mouse hippocampus, intermittent hypoxia altered the expression of many miRNAs, including miR‐448‐3p and miR‐1264‐3p. miR‐448‐3p and miR‐1264‐3p could influence the localization of hnRNPA2B1 by regulating the target gene *Fam76b*. As a splicing factor, hnRNPA2B1 may be involved in the response to intermittent hypoxia by regulating the ratio of *Nbr1* and *Dph3* transcripts.

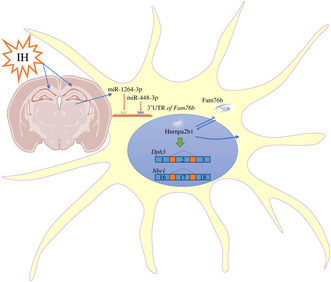

## 1 Introduction

Oxygen is essential for aerobic organisms. The maintenance of oxygen concentration in various tissues of the organism depends on the balance between oxygen delivery to cells and oxygen consumption within cells [1]. To maintain this balance, organisms need to be able to quickly detect changes in oxygen concentration and make adjustments. The fundamental mechanisms of oxygen sensing and control at the cellular level include transcription factors, hypoxia‐inducible factors, prolyl hydroxylase domain (PHD) proteins, and the E3 ubiquitin ligase complex [2]. HIF‐1 is the most important regulator [3].

Hypoxia includes acute hypoxia, chronic hypoxia, and intermittent hypoxia (IH). IH is one of the main pathologies of obstructive sleep apnea syndrome (OSAS) [4]. IH works by causing inflammation and cellular death. In the microglia of rats with intermittent hypoxia, there was an upregulation of IL‐1β, IL‐6, TNFα, and COX‐2 [5]. The Reactive oxygen species (ROS) induced by IH can cause cell death, promote inflammation, and regulate the expression of proinflammatory factors [6, 7].

IH is also associated with Alzheimer's disease (AD). Intermittent hypoxia significantly increases the abnormal deposition of Aβ in the brain of mice, and the accumulation of Aβ is a key mechanism in the pathogenesis of AD [8]. In AD and OSAS patients, continuous positive airway pressure therapy ameliorates IH and enhances language acquisition, memory, and cognitive flexibility [9].

Further study of IH is helpful to provide a theoretical basis for treatment. Cellular responses to hypoxia include transcriptional regulation and post‐transcriptional regulation. HIF plays a key role at the transcriptional level. miRNA and alternative splicing may play an important role in the post‐transcriptional regulation of cellular hypoxic response [10, 11].

In this work, intermittent hypoxia altered the expression of several miRNAs, including miR‐448‐3p and miR‐1264‐3p. miR‐448‐3p and miR‐1264‐3p could influence the localization of hnRNPA2B1 by regulating the target gene *Fam76b*. As a splicing factor, hnRNPA2B1 may be involved in the response to intermittent hypoxia by regulating the ratio of *Nbr1* and *Dph3* transcripts. Our research on the interaction between miRNA and alternative splicing can provide a theoretical basis for the treatment of hypoxia‐related diseases, especially neurodegenerative diseases.

## 2 Materials and Methods

### 2.1 Construction of Intermittent Hypoxia Model Mice

Eight‐week‐old SPF C57BL/6J male mice were purchased from Spfbiotech (China). Mice were housed at a suitable temperature (25°C) and humidity (50%) and fed ad libitum. Before intermittent hypoxia, all mice were weighed (the mice with abnormal weight were removed).

Intermittent hypoxia was performed in a hypoxic chamber (Pro0x‐100, TOW‐INT TECH, China) at 9 a.m. every day for 8 h of cyclic intermittent hypoxia. Each cycle contained 7% oxygen concentration for 2 min and 21% oxygen concentration for 2 min, and the switching between normoxia and hypoxia was completed within 70 s.

Mice were subjected to intermittent hypoxia for 1, 3, 5, or 7 weeks. Mice with intermittent hypoxia for 7 weeks were treated first, and eventually all mice were sampled at the same time. After treatment, the mice were anesthetized. The hippocampus was isolated after dissecting the skull of the mouse. The left hippocampus was fixed in 4% paraformaldehyde or glutaraldehyde fixed solution (PN0019, Pinuofei Biological Technology, China), and the right hippocampus was placed in EP tube and put into liquid nitrogen for subsequent experiments.

### 2.2 Cell Transfection

HT22 hippocampal neuronal cells were purchased from Hunan Fenghui Biotechnology Co. Ltd. (China), and HEK293T cells were stored in our laboratory. In a 37°C, 5% CO_2_ incubator, HT22, and HEK293T cells were cultured in DMEM medium supplemented with 10% fetal bovine serum and 1% penicillin and streptomycin.

Cells were transfected with Lipo6000 (Beyotime, China). For HT22 cells, the cells were seeded in six‐well plates and transfected when the confluence of cells reached 30%–50%. In each well, the amount of miR‐448‐3p or miR‐1264‐3p mimics was 240 pmol. For HEK293T cells, cells were seeded in a 24‐well plate, and transfection was performed when the confluence of cells reached 50%–60%. In each well, the amount of miR‐448‐3p or miR‐1264‐3p mimics was 40 pmol, and the amount of plasmid was 500 ng. Luciferase activity was detected 24 h after transfection.

### 2.3 H&E Staining

HE staining was referred to in the published manuscript [12]. The basic process is as follows: After fixation, the samples were dehydrated with ethanol and cleared with xylene. After paraffin embedding, the tissue was cut into 5‐μm sections. After flotation and drying, the sections were stained with the hematoxylin and eosin staining kit (Beyotime, China) and covered with neutral balsam (Sangon Biotech, China).

### 2.4 Immunohistochemistry and Immunofluorescence

*Immunohistochemistry*: Utilizing the Hypoxyprobe‐1 Plus Kit (HP2‐100 Kit, HPI), the hypoxic status of hippocampus was validated through immunohistochemistry. After 7 h and 25 min of normoxia or intermittent hypoxia, the mice were injected with hypoxia probes (60 μg Hypoxyprobe: 1 g body weight). Then, the treatment was continued under the original conditions for 35 min. Finally, hypoxia probes were detected by immunohistochemistry.

*Immunofluorescence*: The process of immunofluorescence was mentioned in the published manuscript [13]. The basic process is as follows: The treated cells were fixed with 4% paraformaldehyde and permeabilized with Triton X‐100. Goat serum was used for blocking, and anti‐hnRNPA2B1 (1:100) (K002317P, Solarbio, China) was used for hybridization. Utilized a laser scanning confocal microscope to observe and take pictures.

### 2.5 Transmission Electron Microscopy (TEM)

Cardiac perfusion was performed after the mice were anesthetized. The brain was isolated and fixed with 2.5% glutaraldehyde. After all mice were sampled, the brain was trimmed to 1–3 mm^3^ and continued to be fixed in 2.5% glutaraldehyde at room temperature for 2 h, then stored at 4°C. Subsequently, the tissues were dehydrated, embedded, cured with epoxy resins, sectioned, stained, and finally observed and photographed by electron microscopy.

### 2.6 Quantitative Reverse Transcription Polymerase Chain Reaction (RT‐qPCR)

Total RNA was extracted using the RNAiso Plus (Takara Bio, Japan). The mRNA was reverse transcribed into cDNA using the PrimeScript RT reagent kit (Takara Bio, Japan). Using M‐MLV reverse transcriptase (Promega, WI, USA), the miRNAs were reverse transcribed by stem‐loop [14]. The reagent used for qPCR was TB Green Premix Ex Taq II (TaKaRa, Japan). The reverse transcription primers and qPCR primers for miR‐448‐3p and miR‐1264‐3p were designed by sRNAPrimerDB [15]. The reverse transcription primers and qPCR primers are as follows:

miR‐448‐3p‐RT, 5′‐GTCGTATCCAGTGCAGGGTCCGAGGTATTCGCACTGGATACGACATGGGA‐3′

miR‐1264‐3p‐RT, 5′‐GTCGTATCCAGTGCAGGGTCCGAGGTATTCGCACTGGATACGACACAGGT‐3′

miR‐448‐3p‐qPCR, 5′‐AAGCGACCTTGCATATGTAGGA‐3′ and 5′‐GTCGTATCCAGTGCAGGGT‐3′

miR‐1264‐3p‐qPCR, 5′‐AGCCAGCGCAAATCTTATTTGA‐3′ and 5′‐GTCGTATCCAGTGCAGGGT‐3′.

U6 was used as the reference gene. The U6 reverse transcription and qPCR primers were referred to in the published manuscript [16].

The qPCR primers for genes were designed based on the GenBank database of the National Center for Biotechnology Information (NCBI). β‐actin (*Actb*) was used as the reference gene. The qPCR primers for mRNA are as follows:

hnRNPA2B1, 5′‐CCTTTGGAGAGGAAAAAGAGAGAAA‐3′ and 5′‐GTCTGTGAGCTTTCCCCATTG‐3′

Fam76b, 5′‐GTACCAAGTGTCAGCGTTGT‐3′ and 5′‐CAACTTCCCATCAACTTTCCTTC‐3′

β‐actin, 5′‐TAAAACCCGGCGGCGCA‐3′ and 5′‐ATCCATGGCGAACTGGTGG‐3′

### 2.7 Semiquantitative Reverse Transcription Polymerase Chain Reaction (Semiquantitative RT‐PCR)

The gene sequences were downloaded from the Ensembl database, and primers were designed on the upstream exon and the downstream exon of the skipped exon. The primers were designed using Primer3Plus. RT‐PCR products were separated by polyacrylamide gel electrophoresis and stained with Gel‐Red (Beyotime, China). RT‐PCR products were quantified using ImageJ.

The exon inclusion rate was calculated using the formula:

$$\begin{array}{c} \text{Exon Inclusion Rate}=\left(V_{\text{included}}/N_{\text{included}}\right)/\\ \;\left(V_{\text{included}}/N_{\text{included}}+V_{\text{skipped}}/N_{\text{skipped}}\right). \end{array}$$

*V*, Quantitative results of RT‐PCR products; *N*, The number of nucleic acids contained in RT‐PCR products; included, Transcript containing the alternative exon; skipped, Transcript without the alternative exon.

The primer sequences are as follows:

*Dph3*, 5′‐CGGAGACATATTTCTACCCTTGC‐3′ and 5′‐TTGTTGGTTGAAGGTGCTGG‐3′

*Nbr1*, 5′‐GCTGCTGGATATAAACATTGTCC‐3′ and 5′‐GGGATCGAATTACAGTCTCACA‐3′

*Ep400*, 5′‐ACAGAAGCCGACCCCTTTAA‐3′ and 5′‐GTGGTGGGGTATGCAAGCT‐3′.

### 2.8 Western Blot

*Total protein extraction*: Used RIPA buffer (Beyotime, China) containing PMSF to lyse the hippocampus or HT22 cells. After completing the lysis, the total protein was obtained by centrifugation.

*Nuclear protein extraction*: Used the nuclear and cytoplasmic protein extraction kit (Sangon Bioteck, China) for nuclear protein extraction. The basic procedure involves first isolating the nucleus, followed by lysis of the nucleus, and finally separating the nuclear proteins by centrifugation.

The protein samples were stored in the ultra‐low‐temperature refrigerator.

*Electrophoresis and protein transfer*: After adding loading buffer (YangGuangBio, China), protein samples were denatured at 99°C for 10 min. Then, SDS polyacrylamide gel electrophoresis was performed. Finally, the proteins were transferred from the gel to the PVDF membrane.

*Blotting*: Membranes were incubated in 5% non‐fat dry milk at room temperature for 2 h. PVDF membranes were then incubated overnight at 4°C with the corresponding antibodies:

β‐actin antibody (1:50,000) (AC026, ABclonal, China), FAM76B antibody (1:2000) (A303‐369A‐T, Thermo Fisher, MA, USA), hnRNPA2B1 antibody (1:1000) (K002317P, Solarbio, China), and H3 antibody (1:5000) (PTM‐6621, PTM Bio, China).

Finally, PVDF membranes were incubated with goat anti‐rabbit IgG (1:15,000) (ZB‐2301, ZSBio, China) for 1 h at room temperature. Proteins were detected by ECL (Beyotime, China). The protein bands were quantified by ImageJ.

### 2.9 Vector Construction and Luciferase Assay

The construction of the psiCHECK‐2 vectors and luciferase assay were referred to in the manuscript [17], and the basic procedure is described below. The fragments containing the predicted binding sites of miR‐448‐3p and miR‐1264‐3p in the 3′UTR of the mouse *Fam76b* gene were amplified by PCR. These fragments were individually cloned into the psiCHECK‐2 vector. The predicted binding sites for miR‐448‐3p and miR‐1264‐3p on these fragments were mutated using fusion PCR. The mutated sequences were also cloned into the psiCHECK‐2 vector. The regulation of miR‐448‐3p and miR‐1264‐3p on the corresponding sequences was determined by comparing the relative activities of Renilla luciferase and Firefly luciferase.

### 2.10 Data Source

By RNA sequencing, previous research has examined the differential alternative splicing events following hnRNPA2B1 suppression [18]. https://www.ncbi.nlm.nih.gov/pmc/articles/PMC5123850/bin/NIHMS823425‐supplement‐2.xlsx.

Differentially expressed genes (|log_2_FC| > 1 & *p*‐adjust < 0.05) and differential alternative splicing events (*p*‐value < 0.05) induced by intermittent hypoxia were downloaded from https://1drv.ms/u/c/c12108d59b164800/EcBf8CuJFA5JocKfgFdSOCMBl7V8mXzxKt5GJwBYCWiucQ?e=hkUn3D.

### 2.11 Statistical Analysis

The data were analyzed for normality before statistical analysis of the results of RT‐qPCR and semiquantitative PCR. When the data met the assumption of normality, the Student's *t*‐test was used for statistical analysis of RT‐qPCR and semiquantitative PCR, and *p* < 0.05 was statistically significant. *p < 0.05; **p < 0.01. When the data did not meet the assumption of normality, we used the Mann–Whitney *U*‐test for statistical analysis. The data analyzed by the Mann–Whitney *U*‐test were illustrated in the figure legend. The Venn diagram was drawn by Jvenn and VENNY2.1. miRNA target genes were predicted by the miRWalk, TargetScanMouse8.0, and miRDB databases. The phylogenetic tree was constructed using the MEGA 11.0 software.

## 3 Results

### 3.1 Intermittent Hypoxia‐Induced Hippocampal Damage in Mice

**FIGURE 1:**
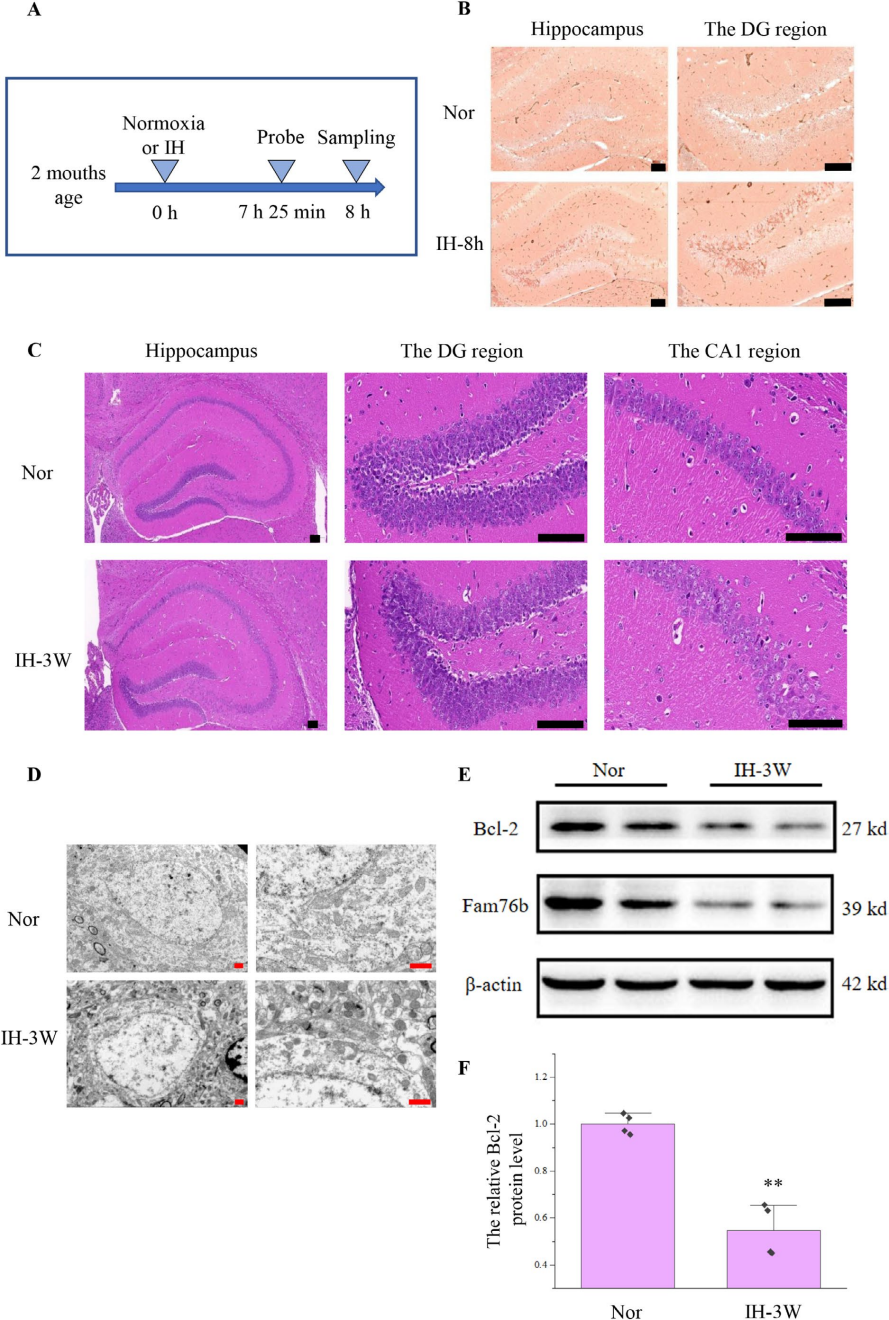
Intermittent hypoxia caused hippocampal damage. Nor: Normoxia. IH‐8 h: 8 h of intermittent hypoxia. IH‐3 W: 3 weeks of intermittent hypoxia. (A) Schematic diagram of hypoxia probe addition and detection. (B) The hypoxia probe was detected by immunohistochemistry. The scale bar represents 100 μm, and the brown areas indicate hypoxia probe. (C) He staining was used to detect the hippocampus after 3 weeks of intermittent hypoxia. The scale bar represents 100 μm. (D) The hippocampus was detected by transmission electron microscopy. The scale bar represents 10 μm. (E) Western blot was used to detect the expression of Bcl‐2. (F) Quantification of the protein level shown in (E). *N* = 4, **p < 0.01.

Using the hypoxia probe, we first examined whether intermittent hypoxia (7% O_2_) might cause hypoxia in the hippocampus of mice (Figure 1A). The results of immunohistochemistry revealed that numerous cells in the hippocampus (especially in the DG region) of mice under intermittent hypoxia were stained brown (Figure 1B). It is proved that intermittent hypoxia (7% O_2_) could cause hypoxia in the hippocampus.

In order to further elucidate the effect of intermittent hypoxia, the brain was examined by HE staining. There was no obvious abnormality in the hippocampus of mice after intermittent hypoxia for 1 week (Figure S1). After 3 weeks of hypoxia, the pyramidal cells were loosely distributed, and the cell contour was not clear (Figure 1C).

Subsequently, the hippocampus was detected by transmission electron microscopy (Figure 1D). Mitochondria in hippocampal cells increased after intermittent hypoxia. At the same time, the mitochondria became darker due to shrinkage, and some mitochondria were ruptured. Therefore, the damage caused by intermittent hypoxia may be related to mitochondrial abnormalities and apoptosis.

The Bcl‐2 downregulation is closely related to mitochondrial abnormalities, apoptosis, and Alzheimer's disease [19]. So we detected the expression of Bcl‐2 in the hippocampus after intermittent hypoxia. Western blot showed that (Figure 1E,F), compared with normoxia, the expression of Bcl‐2 decreased by 45% after hypoxia.

### 3.2 Screening of Key miRNAs and Target Genes

**FIGURE 2:**
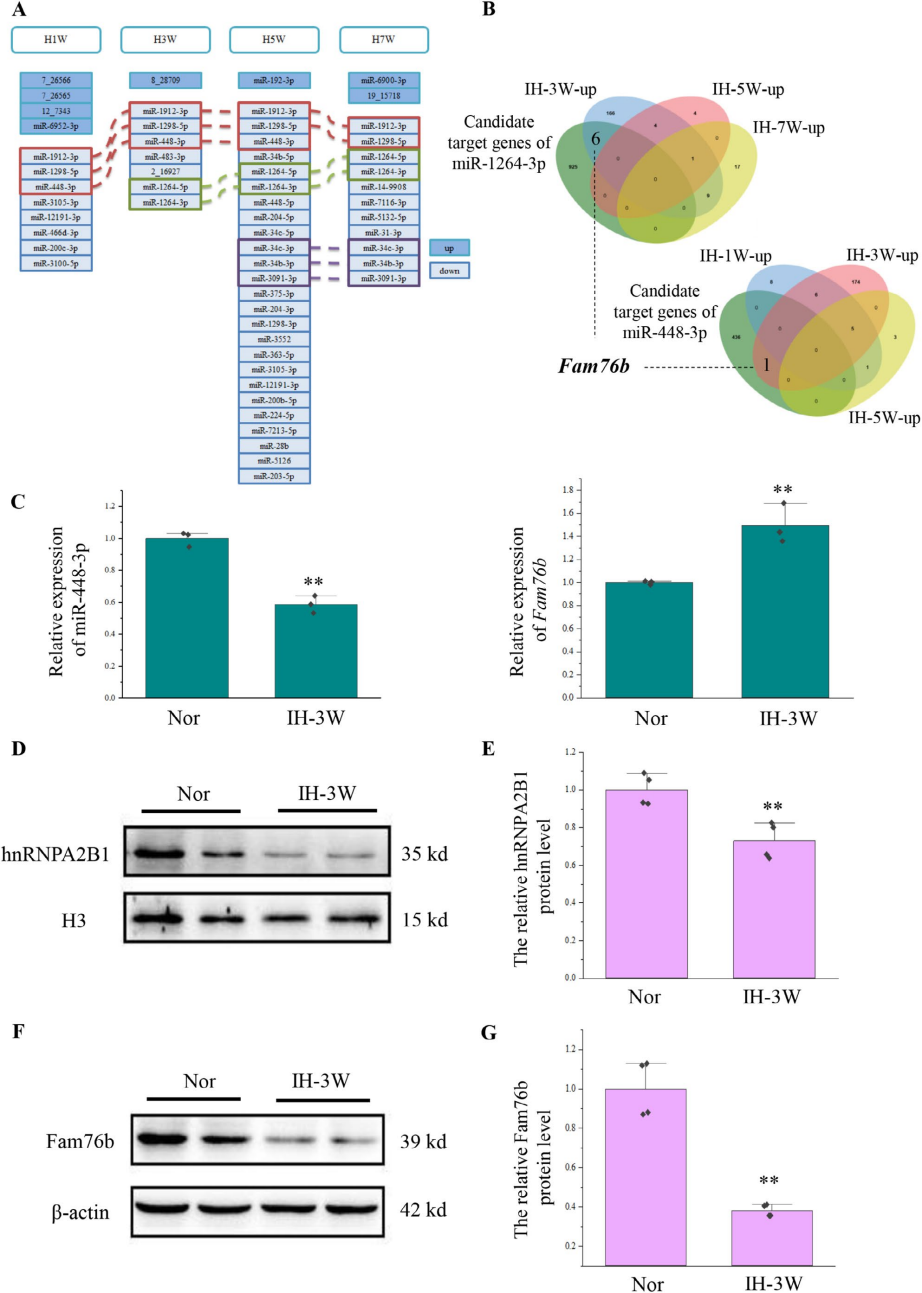
Screening and validation of key miRNAs. Nor: Normoxia. IH‐XW: X week(s) of intermittent hypoxia. (A) Differentially expressed miRNAs induced by intermittent hypoxia (*p*‐value < 0.05 & |log_2_FC| > 1). The red box represents the first category of miRNA, the green box represents the second category of miRNA, and the purple box represents the third category of miRNA. (B) Analysis of target genes that may be regulated by miR‐448‐3p and miR‐1264‐3p during intermittent hypoxia. (C) RT‐qPCR was used to detect the expression of miR‐448‐3p and *Fam76b* in the hippocampus after intermittent hypoxia for 3 weeks. The reference genes were U6 or β‐actin (*Actb*). *N* = 3, **p < 0.01. (D) Western blot was used to detect the expression of hnRNPA2B1 in the nucleus of the hippocampus after intermittent hypoxia for 3 weeks. The reference protein was H3. (E) Quantification of the hnRNPA2B1 proteins, *N* = 4, **p < 0.01. (F) Western blot was used to detect the expression of Fam76b in the hippocampus after intermittent hypoxia for 3 weeks. The reference protein was β‐Actin. (G) Quantification of the Fam76b proteins, *N* = 4, **p < 0.01.

In order to study the key miRNAs, the miRNA sequencing of the hippocampus was performed after 1 week, 3 weeks, 5 weeks, and 7 weeks of intermittent hypoxia. Based on the miRNA sequencing results, the differentially expressed miRNAs were screened according to *p*‐value < 0.05 and |log_2_FC| > 1. The analysis of miRNAs at various time points after intermittent hypoxia showed that the differentially expressed miRNAs could be divided into three categories (Figure 2A). The first category of miRNA, including mmu‐miR‐448‐3p, mmu‐miR‐1912‐3p, and mmu‐miR‐1298‐5p, was changed after 1, 3, and 5 weeks of intermittent hypoxia. The expression of mmu‐miR‐1912‐3p and mmu‐miR‐1298‐5p also changed after 7 weeks of intermittent hypoxia. The second category was miRNA whose expression was changed after 3, 5, and 7 weeks of intermittent hypoxia, including mmu‐miR‐1264‐3p and mmu‐miR‐1264‐5p. The third category included mmu‐miR‐34c‐3p, mmu‐miR‐34b‐3p, and mmu‐miR‐3091‐3p, they were altered at 5 and 7 weeks of intermittent hypoxia. All these miRNAs were downregulated after intermittent hypoxia.

We conducted subsequent analysis on these three categories of miRNAs. miRNA usually achieves its function by inhibiting the expression of target genes. We predicted and compared the target genes of the above three categories of miRNA through three databases (miRWalk, TargetScan, and miRDB) (Figure S2). The genes analyzed in two or more databases were used as the candidate target genes for subsequent analysis (Table S1). Based on transcriptome sequencing results from the mouse hippocampus at 1, 3, 5, and 7 weeks of intermittent hypoxia (Materials and methods, 2.10 Data Source), we analyzed the genes that may be regulated by miRNAs during this process. We obtained the predicted target genes by comparing the candidate target genes of miRNAs with the upregulated differentially expressed genes induced by intermittent hypoxia (Figure S3; Figure 2B). Among them, although the expression of mmu‐miR‐1298‐5p was changed after 1, 3, 5, and 7 weeks of hypoxia stress, there was no candidate target gene of mmu‐miR‐1298‐5p among the up‐regulated differentially expressed genes at these four time points. Similarly, the expression of mmu‐miR‐3091‐3p was changed after 5 and 7 weeks of hypoxia stress, but there was no candidate target gene of mmu‐miR‐3091‐3p among the up‐regulated differentially expressed genes at these two time points (Figure S3).

Apart from mmu‐miR‐1298‐5p and mmu‐miR‐3091‐3p, the other miRNAs in these three categories had at least one predicted target gene at the time point when their expression was changed. Interestingly, miR‐448‐3p and miR‐1264‐3p, whose expression changed after 3 weeks of hypoxia stress, shared a common predicted target gene, namely *Fam76b* (Figure 2B). Based on the results of HE staining and transmission electron microscopy, we have found structural abnormalities in hippocampal cells after 3 weeks of hypoxia stress. At the same time, Fam76b is closely related to neuroinflammation [20, 21]. Moreover, target gene analysis revealed that miR‐448‐3p and miR‐1264‐3p could jointly regulate the expression of *Fam76b* under intermittent hypoxia for 3 weeks. Therefore, we chose to conduct a follow‐up research on the regulation of Fam76b by miR‐448‐3p and miR‐1264‐3p and their functions under intermittent hypoxia for 3 weeks.

Firstly, the expression of miR‐448‐3p, miR‐1264‐3p, and *Fam76b* in the mouse hippocampus undergoing intermittent hypoxia for 3 weeks was verified by RT‐qPCR (Figure 2C). However, the expression of miR‐1264‐3p was too low to be detected by RT‐qPCR. Consistent with sequencing data, intermittent hypoxia caused the downregulation of miR‐448‐3p and the upregulation of *Fam76b*.

Next, we explored the function of *Fam76b* during intermittent hypoxia. Published manuscripts have shown that FAM76B can affect neuroinflammation by promoting hnRNPA2B1 nuclear import [21]. Therefore, the expression of hnRNPA2B1 in the nucleus (Figure 2D,E) and in the cell (Figure S4) was detected. The results showed that hnRNPA2B1 decreased in the nucleus, which was inconsistent with the increase in mRNA levels of *Fam76b*. To better elucidate the expression of *Fam76b*, Western blot was used (Figure 2F,G). Western blot showed that intermittent hypoxia caused Fam76b downregulation at the protein level.

### 3.3 Screening of Differential Alternative Splicing Events Regulated by hnRNPA2B1

**FIGURE 3:**
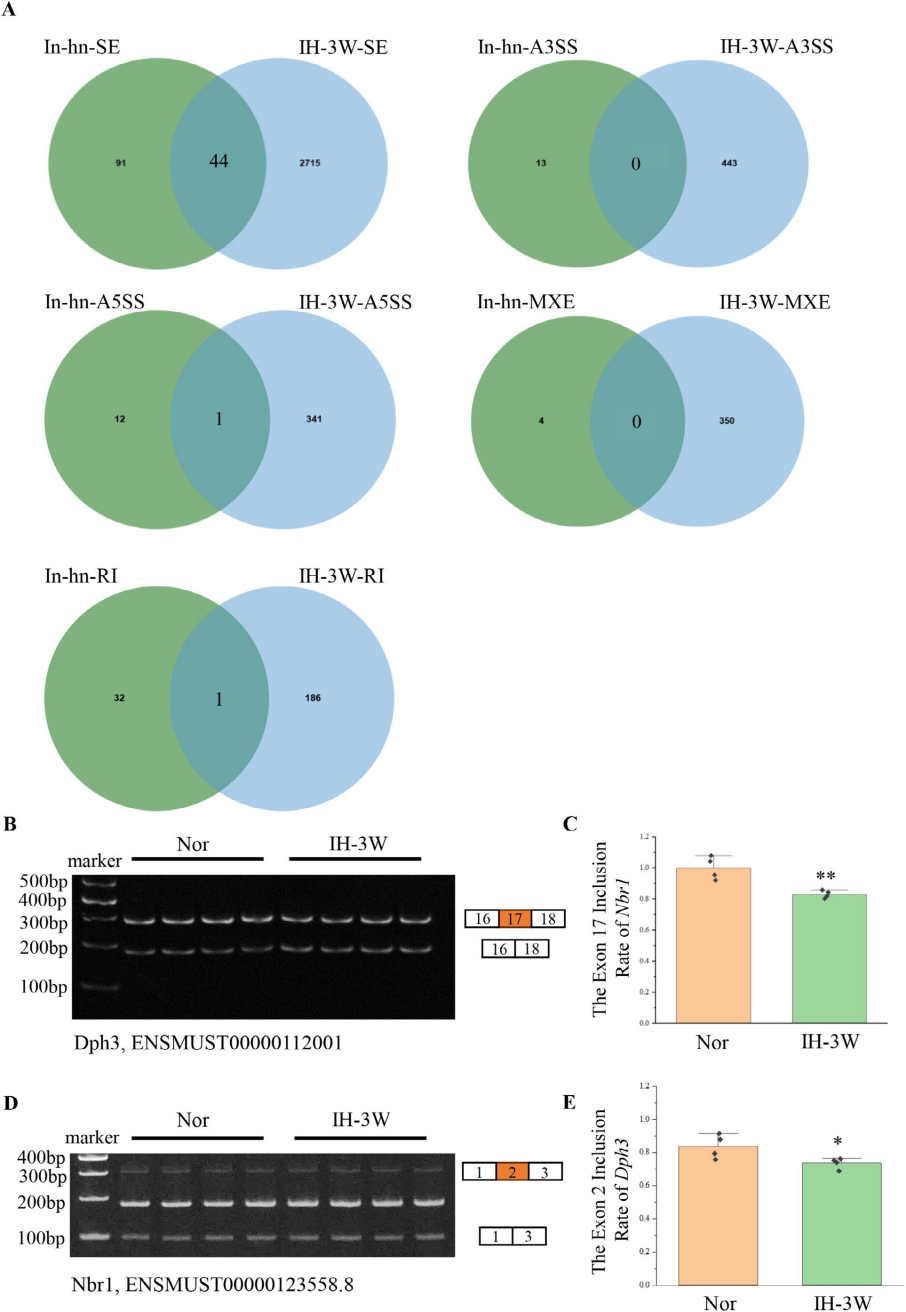
Screening and validation of alternative splicing events regulated by hnRNPA2B1. (A) Venn diagram analysis of alternative splicing events that may be regulated by hnRNPA2B1 under intermittent hypoxia. In‐hn: Genes with differential alternative splicing after inhibition of hnRNPA2B1. IH‐3 W: Genes with differential alternative splicing after 3 weeks of intermittent hypoxia. SE: Skipped exon; RI: Retained intron; MXE: Mutually exclusive exons; A5SS: Alternative 5′ splice site; A3SS: Alternative 3′ splice site. (B) Exon inclusion of *Nbr1* was detected by semiquantitative PCR. (C) Quantification of the transcripts in (B) and calculation of the ratio of exon inclusion of *Nbr1*, *N* = 4, **p < 0.01. (D) Exon inclusion of *Dph3* was detected by semiquantitative PCR. (E) Quantification of the transcripts in (D) and calculation of the ratio of exon inclusion of *Dph3*, *N* = 4, *p < 0.05.

Alternative splicing plays an important role in neurogenesis and brain development. Aberrant splicing can lead to brain diseases [22]. The translocation of hnRNPA2B1 affects alternative splicing. Based on the RNA sequencing results in our previous study (Materials and methods, 2.10 Data source), 3 weeks of intermittent hypoxia induced 2574 differential alternative splicing events (Figure S5). Previous studies have analyzed the inhibition of hnRNPA2B1 induced differential alternative splicing events by RNA sequencing [18]. The genes with differential alternative splicing induced by intermittent hypoxia and the genes with differential alternative splicing regulated by hnRNPA2B1 were analyzed by Venn diagram (Figure 3A). Under intermittent hypoxia, hnRNPA2B1 may regulate the alternative 5′ splice site of *Fbxl4*, the retained intron of *Gas5*, and the skipped exon of 44 genes. Further analysis of these 46 genes showed that 13 genes had the same alternative splicing site after intermittent hypoxia and hnRNPA2B1 inhibition. These genes were screened and ranked according to the △PSI and the reads (the mean of reads > 10). The top three differential alternative splicing events (corresponding genes were *Nbr1*, *Dph3*, and *Ep400*) all belonged to skipped exon.

These three genes were detected by semiquantitative PCR. The exon inclusion of *Nbr1* and *Dph3* was decreased by intermittent hypoxia (Figure 3B–E), which was consistent with the results of RNA sequencing. However, no changes in the exon inclusion of *Ep400* were detected (Figure S6). The SC2disease database was used to analyze the association of *Nbr1* and *Dph3* with neurological diseases. The results showed that the expression of these two genes changed in various diseases (Figure S7). In response to intermittent hypoxia, the nuclear export of hnRNPA2B1 may affect the alternative splicing of genes including *Nbr1* and *Dph3*.

### 3.4 Functional Study of miR‐448‐3p and miR‐1264‐3p

**FIGURE 4:**
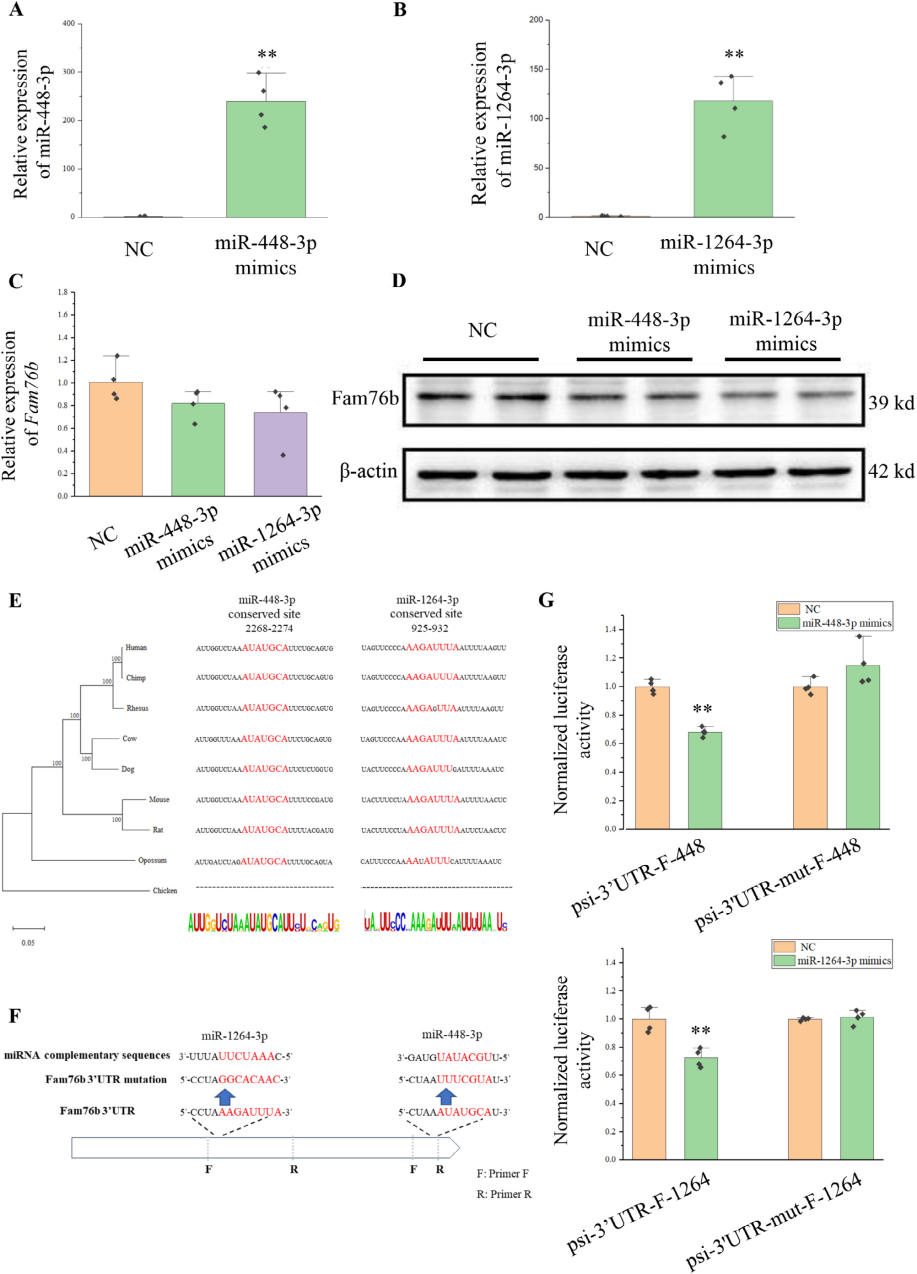
*Fam76b* is the target gene of miR‐448‐3p and miR‐1264‐3p. (A, B) RT‐qPCR was used to verify the expression of miR‐448‐3p and miR‐1264‐3p 12 h after mimics transfection. U6 was used as the reference gene. *N* = 4. **p < 0.01. (C) RT‐qPCR was used to verify the expression of *Fam76b* 12 h after mimics transfection. β‐actin was used as the reference gene. *N* = 4. (D) The expression of Fam76b was detected by Western blot 24 h after mimics transfection. β‐actin was used as the reference protein. (E) The phylogenetic tree was constructed based on the 3′UTR of *Fam76b* across multiple species. The complementary sites of miR‐448‐3p and miR‐1264‐3p and flanking sequences in the 3′UTR of *Fam76b* of multiple species were analyzed. (F) Illustration of PCR amplification and mutation of the 3′UTR fragments of *Fam76b*. (G) After co‐transfection of miRNA mimics with recombinant vector (psi‐3′UTR‐F‐448, psi‐3′UTR‐F‐1264, psi‐3′UTR‐mut‐F‐448, or psi‐3′UTR‐mut‐F‐1264) for 24 h, the activities of Renilla luciferase and Firefly luciferase were detected. *N* = 4, **p < 0.01.

Next, we studied the effect of miR‐448‐3p and miR‐1264‐3p on the expression of Fam76b at the cellular level. miRNA mimics were transfected into HT22 cells, and overexpression of miR‐448‐3p and miR‐1264‐3p was determined by RT‐qPCR (Figure 4A,B). Using qPCR and Western blot, the expression of Fam76b was detected. Results demonstrated that miR‐448‐3p and miR‐1264‐3p mimics could inhibit Fam76b (Figure 4C,D).

We verified whether miR‐448‐3p and miR‐1264‐3p can directly act on the complementary sites of the 3′UTR of *Fam76b*. By constructing a phylogenetic tree, we analyzed the 3′UTR of *Fam76b* across multiple species (Figure 4E). The phylogenetic tree was consistent with the morphological taxonomy. In mammals, these two complimentary sites were highly conserved, but lacking in chickens.

We detected the regulatory effects of miR‐448‐3p and miR‐1264‐3p on complementary sites using the psiCHECK‐2 vector. The fragment containing miR‐448‐3p or miR‐1264‐3 complementary sites on the 3′UTR of *Fam76b* was cloned into the psiCHECK‐2 vector, namely psi‐3′UTR‐F‐448 or psi‐3′UTR‐F‐1264. Fragments with mutations at complementary sites were also cloned into psiCHECK‐2 vectors, namely psi‐3′UTR‐mut‐F‐448 or psi‐3′UTR‐mut‐F‐1264 (Figure 4F).

The vector and miRNA mimics were co‐transfected into HEK293T cells, and the relative activity of luciferase was detected 24 h after transfection. When miRNA mimics were co‐transfected with psi‐3′UTR‐F‐448 or psi‐3′UTR‐F‐1264, the relative activity of the Renilla luciferase was reduced (Figure 4G). However, when miRNA mimics were co‐transfected with psi‐3′UTR‐mut‐F‐448 or psi‐3′UTR‐mut‐F‐1264, the relative activity of Renilla luciferase was not changed. The above results indicated that both miR‐448‐3p and miR‐1264‐3p had direct effects on the complementary sites of the 3′UTR of *Fam76b*.

**FIGURE 5:**
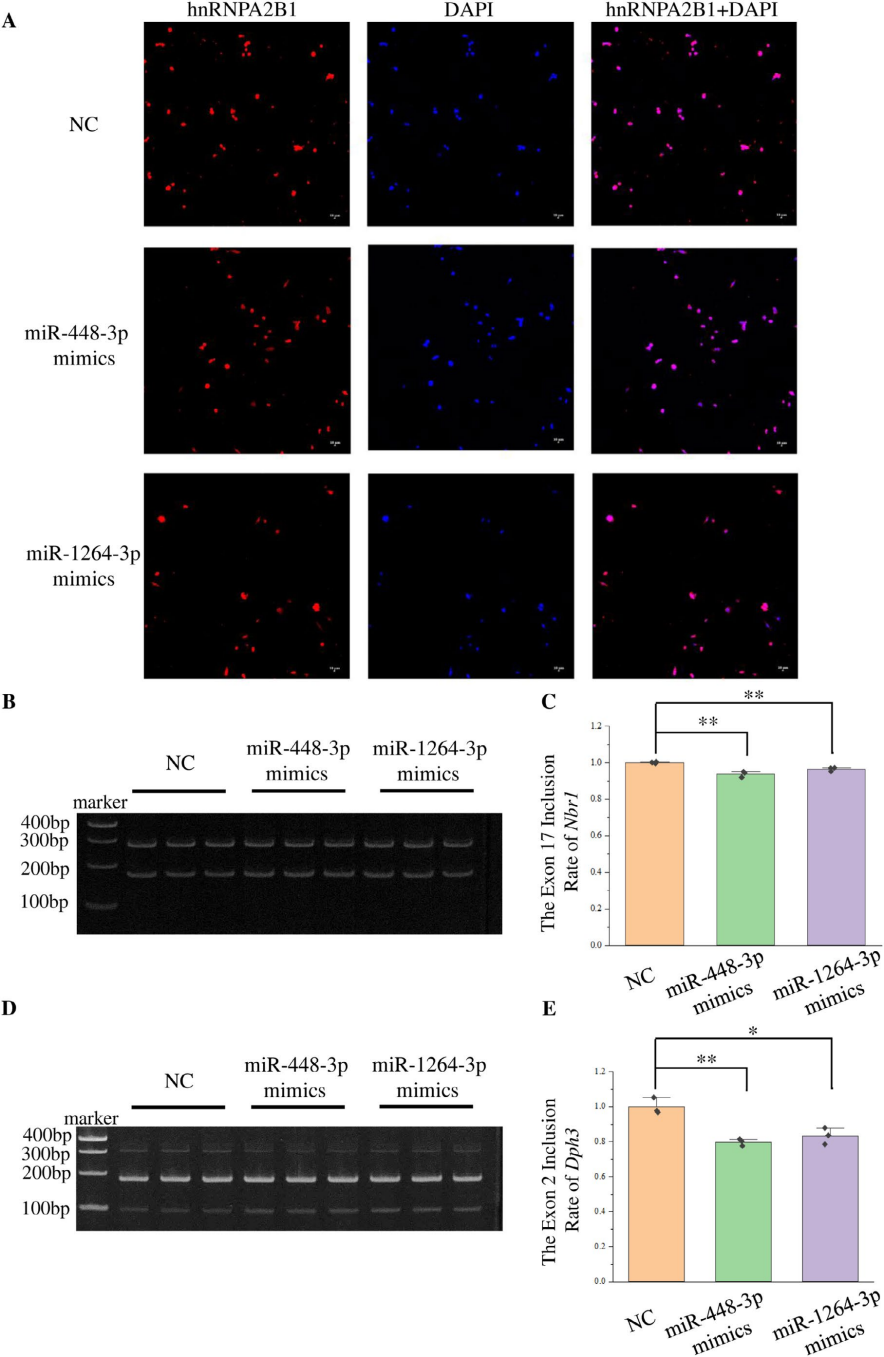
miRNA mimics transfection affected the intracellular distribution of hnRNPA2B1 and the ratio of *Nbr1* and *Dph3* transcripts. (A) After 36 h of mimics transfection, the localization of hnRNPA2B1 in cells was detected by immunofluorescence. Blue represents DAPI staining. Red represents the hnRNPA2B1. The scale bar represents 10 μm. (B) After mimics transfection for 48 h, exon inclusion of *Nbr1* was detected by semiquantitative PCR. (C) Quantification of the transcripts in (B) and calculation of the ratio of exon inclusion of *Nbr1*. *N* = 3, **p < 0.01. (D) After mimics transfection for 48 h, exon inclusion of *Dph3* was detected by semiquantitative PCR. (E) Quantification of the transcripts in (D) and calculation of the ratio of exon inclusion of *Dph3*. *N* = 3, *p < 0.05, **p < 0.01.

Could the regulation of miR‐448‐3p and miR‐1264‐3p on *Fam76b* influence the ectopic expression of hnRNPA2B1 in cells and lead to the change of alternative splicing? Therefore, after transfection of miR‐448‐3p or miR‐1264‐3p mimics, the intracellular localization of hnRNPA2B1 was detected by immunofluorescence. Overexpression of miRNA promoted the distribution of hnRNPA2B1 in the cytoplasm (Figure 5A; Figure S8). The overexpression of miRNA decreased the exon inclusion of *Nbr1* and *Dph3*, as evidenced by the detection of alternative splicing (Figure 5B–E).

The findings indicate that overexpression of miR‐448‐3p and miR‐1264‐3p enhanced the translocation of hnRNPA2B1 from the nucleus into the cytoplasm and lowered the exon inclusion of *Dph3* and *Nbr1*. In order to verify that the change of the exon inclusion caused by miRNA was associated with hnRNPA2B1, hnRNPA2B1 was inhibited by siRNA.

**FIGURE 6:**
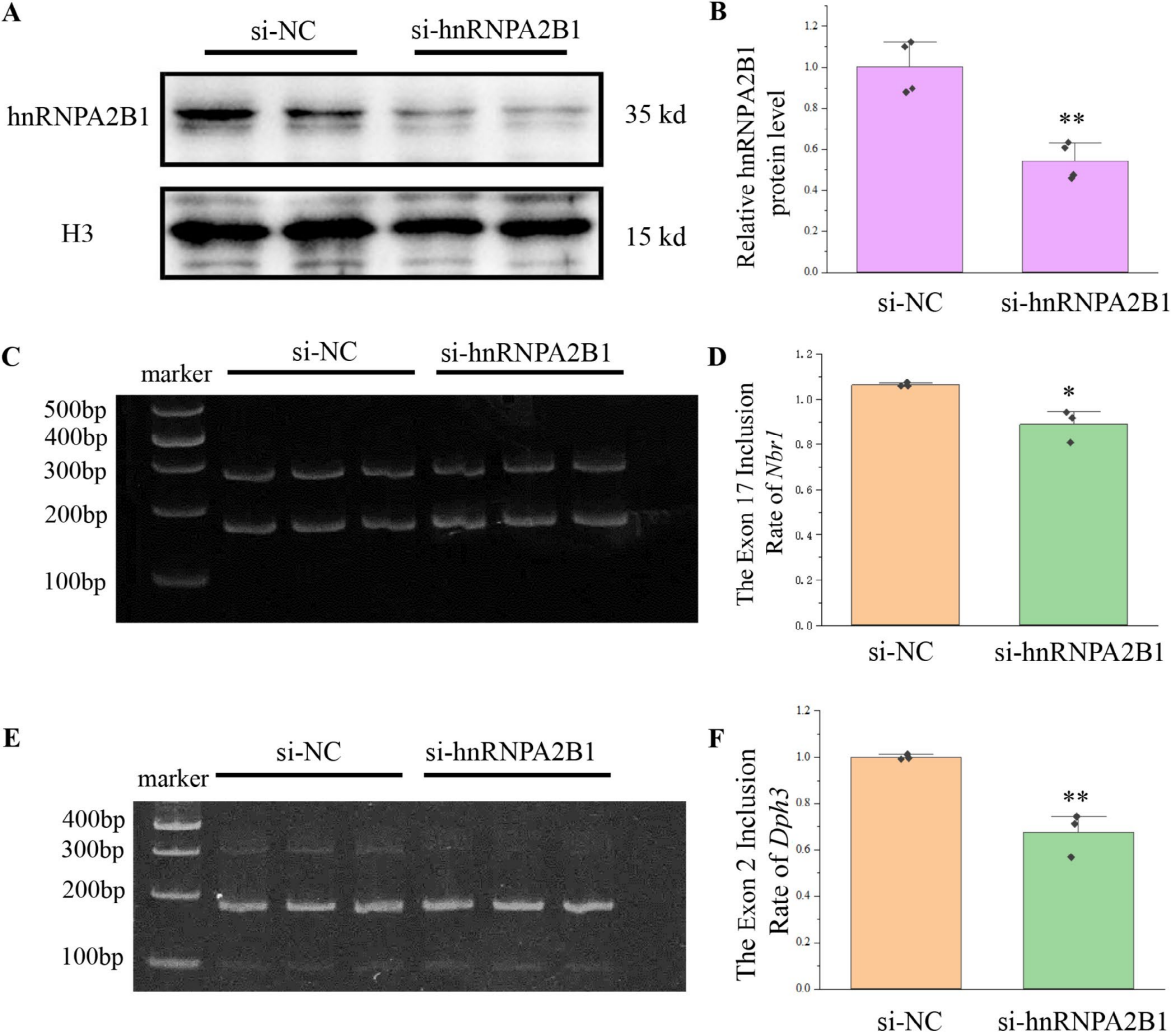
Knockdown of hnRNPA2B1 affected the exon inclusion of *Dph3* and *Nbr1*. (A) Western blot was used to detect the expression of hnRNPA2B1 in the nucleus after transfection of hnRNPA2B1 siRNA for 24 h. H3 was used as the reference protein. (B) Quantification of the hnRNPA2B1 proteins. *N* = 4, **p < 0.01. (C) After transfection of hnRNPA2B1 siRNA for 36 h, exon inclusion of *Nbr1* was detected by semiquantitative PCR. (D) Quantification of the transcripts in (C) and calculation of the exon inclusion of *Nbr1*. *N* = 3, **p < 0.01. (E) After transfection of hnRNPA2B1 siRNA for 36 h, exon inclusion of *Dph3* was detected by semiquantitative PCR. (F) Quantification of the transcripts in (E) and calculation of the exon inclusion of *Dph3*. *N* = 3, **p < 0.01.

After confirming that hnRNPA2B1 was inhibited (Figure 6A,B), the transcripts of *Dph3* and *Nbr1* were detected. The exon inclusion of *Nbr1* and *Dph3* was reduced upon inhibition of hnRNPA2B1 (Figure 6C–F), which is consistent with the reduction in the nucleus of hnRNPA2B1. Therefore, miR‐448‐3p and miR‐1264‐3p can affect the alternative splicing of *Nbr1* and *Dph3* by regulating Fam76b and the localization of the splice factor hnRNPA2B1.

## 4 Discussion

As a key pathogenic factor of obstructive sleep apnea syndrome, intermittent hypoxia can cause a variety of diseases, especially neurodegenerative diseases. However, compared with acute hypoxia and chronic hypoxia, intermittent hypoxia research is very limited. In this study, miRNA and alternative splicing in intermittent hypoxia were studied by establishing animal models, miRNA sequencing, bioinformatics analysis, and functional research.

miRNA plays a crucial regulatory role in Alzheimer's disease. For example, overexpression of miR‐106a and miR‐520c can suppress the Amyloid precursor protein [23], while miR‐29 can influence the activity of BACE1 and the production of Aβ [24]. The changes of amyloid precursor protein and the production of Aβ are the key factors in the occurrence and development of Alzheimer's disease. Alzheimer's disease can also be influenced by splicing factors. For example, a reduction in the splicing factor hnRNPA2B1 may result in an increase in the *BACE1* isoform and encourage the accumulation of Aβ plaques [25]. Our exploration of the relationship between miRNA and alternative splicing in Alzheimer's disease has brought a new perspective to the study of this disease.

In this study, the expression of Fam76b in the mouse hippocampus with intermittent hypoxia for 3 weeks was detected, and it was found that Fam76b was upregulated at the mRNA level and downregulated at the protein level. It is not uncommon in biological research that the expression of mRNA and protein is inconsistent. This may be caused by a variety of regulatory mechanisms, such as post‐transcriptional regulation, post‐translational modifications, changes in mRNA stability and translation efficiency, and feedback regulation. The regulation of *HIF1A* is an example. In normal oxygen concentrations, although *HIF1A* mRNA is transcribed and translated, prolyl hydroxylase domain (PHD) will promote the degradation of HIF‐1α through hydroxylation. Hypoxia does not cause a dramatic change in *HIF1A* mRNA, but because hypoxia inhibits the activity of PHD, PHD will not be able to hydroxylate HIF‐1α. This leads to a rapid increase in HIF‐1α protein [3]. Therefore, a variety of factors may cause inconsistencies between mRNA and protein.

Alternative splicing is crucial for both normal development and disease incidence, and splicing factors are key regulators in the process of alternative splicing. Screening of key splicing factors is usually based on RNA‐seq to identify the differentially expressed splicing factors at the mRNA level [26]. This study examined the effect of hypoxia on hnRNPA2B1 in the nucleus. In addition, it was found that hnRNPA2B1 may respond to hypoxia by regulating the ratio of Dph3 and Nbr1 transcripts. Therefore, screening only by RNA‐seq is not good enough, and detection of changes in protein distribution by proteomics will be an important content of subsequent studies.

This study found that miR‐448‐3p and miR‐1264‐3p could regulate Fam76b. The downregulation of Fam76b promoted the translocation of hnRNPA2B1 from the nucleus to the cytoplasm. Published manuscripts have demonstrated that hnRNPA2B1 can promote the accumulation of Aβ plaques by affecting the ratio of *BACE1* isomers [25]. Our investigation revealed that hnRNPA2B1 regulated the alternative splicing of the *Dph3* and *Nbr1* genes. Dph3 and Nbr1 are closely related to neural development and neurological diseases. Dph3 is involved in the tRNA wobble uridine modification (5‐carboxymethyl‐2‐thiouridine) [27] and the synthesis of diphthamide [28]. Diphthamide is a unique protein post‐translational modification on eukaryotic elongation factor 2 (eEF2). Mutations in eEF2 can induce various types of neural development disorders [29]. Meanwhile, knockout of *Dph3* in mice leads to increased degeneration and necrosis of the neural tube during embryonic development [30]. Nbr1 can interact with p62 to selectively recognize ubiquitinated proteins and organelles [31]. Studies have found that Nbr1 is highly expressed in a variety of neurodegenerative diseases [32], and aberrant regulation of p62 has been shown to cause multiple neurodegenerative diseases [33, 34]. The analysis based on the SC2disease database also indicates that *Dph3* and *Nbr1* are associated with Alzheimer's disease.

In conclusion, the exploration of miRNA and alternative splicing during intermittent hypoxia can provide a theoretical reference for neurodegenerative diseases, especially Alzheimer's disease.

## Author Contributions

C.L. designed and performed experiments, analyzed data, and wrote the paper; D.Q. performed experiments, analyzed data, and wrote the paper; C.L. performed experiments and revised paper; W.P. performed experiments; J.L. analyzed data; L.C. conceived the study, analyzed the data, and revised the paper. All authors contributed to the final version of the paper.

## Ethics Statement

This study was approved by the Institutional Animal Care and Use Committee of the Inner Mongolia University of Science and Technology (NMGKJDX‐2021‐10‐12).

## Conflicts of Interest

The authors declare no conflicts of interest.

## Supporting information


Figures S1–S8.

Table S1.

## Data Availability

The data that supports the findings of this study are available in the Supporting Information of this article.
