# Supplementary material for: miR‐448‐3p/miR‐1264‐3p Participates in Intermittent Hypoxic Response in Hippocampus by Regulating Fam76b/hnRNPA2B1
Source: CNS Neurosci Ther. 2025 Feb 6;31(2):e70239. doi: 10.1111/cns.70239 (PMC11799915; doi:10.1111/cns.70239)
Supplement: Supplementary file 1 — Figures S1–S8. [file CNS-31-e70239-s002.docx]

**miR-448-3p/miR-1264-3p participate in intermittent hypoxic response in hippocampus by regulating Fam76b/hnRNPA2B1**

Chuncheng Liu ^1,2,#^, Donghui Qu ^1,2,#^, Chaoxun Li ^1^, Wenhua Pu ^1,2^, Jun Li ^1,2^ and Lu Cai ^1,2,^*

1 School of Life Science and Technology, Inner Mongolia University of Science and Technology, Baotou, 014010, China;

2 Inner Mongolia Key Laboratory of Functional Genome Bioinformatics

^#^ These authors contributed equally to this work.

^*^ Correspondence: nmcailu@163.com


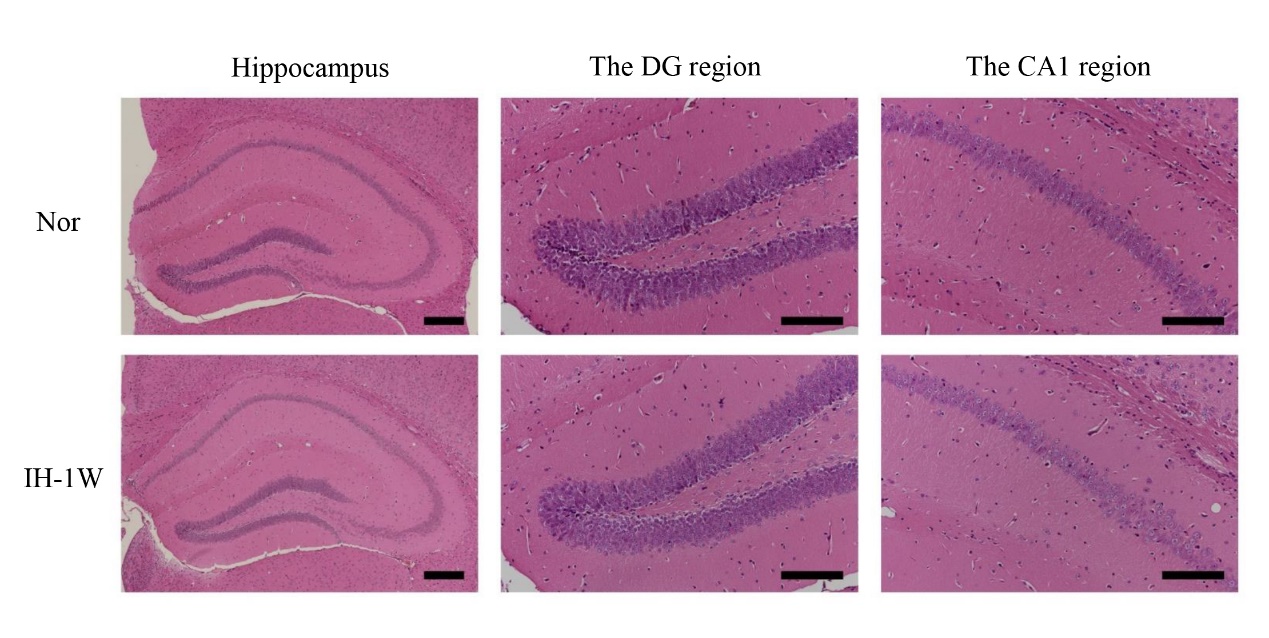
**Supplementary Figure 1 He staining of hippocampus.** He staining was used to detect the hippocampus after 1 week of intermittent hypoxia. The scale bar represents 100 μm. Nor:normoxia. IH-1W: 1 week of intermittent hypoxia.


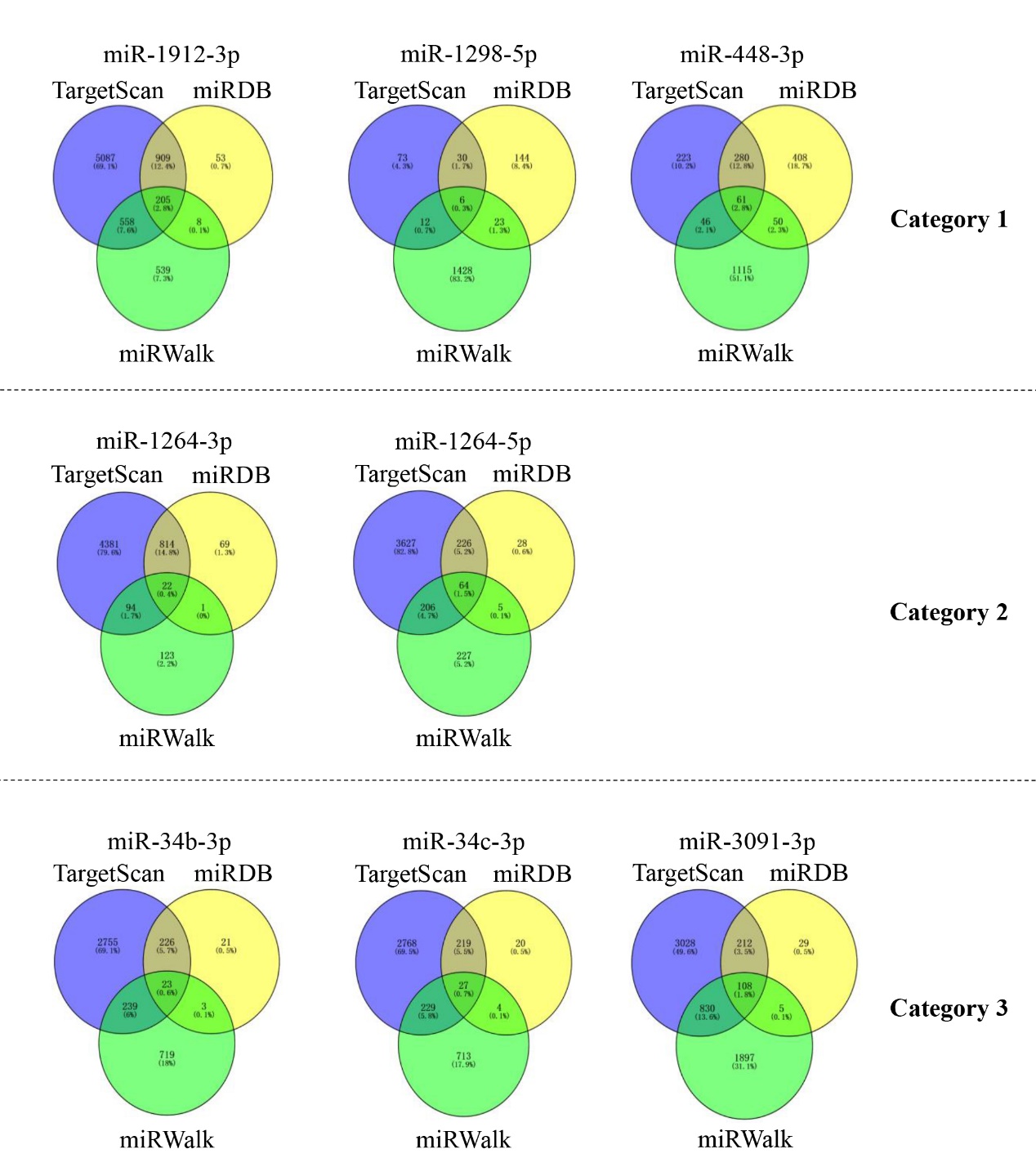
**Supplementary Figure 2 Analysis of target genes of three categories of miRNAs.** miRNA target genes were predicted by the miRWalk, TargetScanMouse8.0, and miRDB databases.


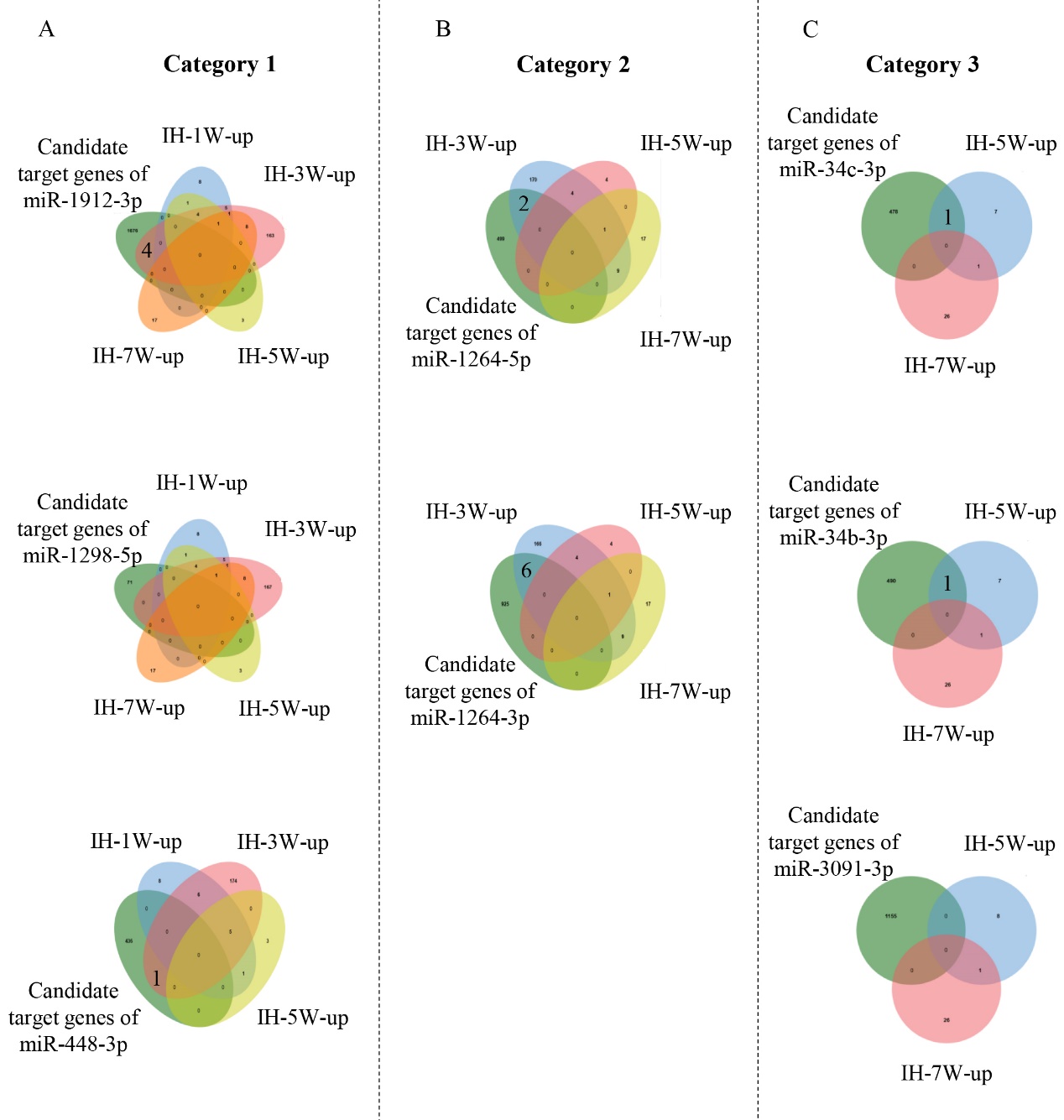
**Supplementary Figure 3 Analysis of target genes of three categories of miRNAs during intermittent hypoxia.** Venn diagram was used to analyze target genes that may be regulated by three categories of miRNAs during intermittent hypoxia.

**
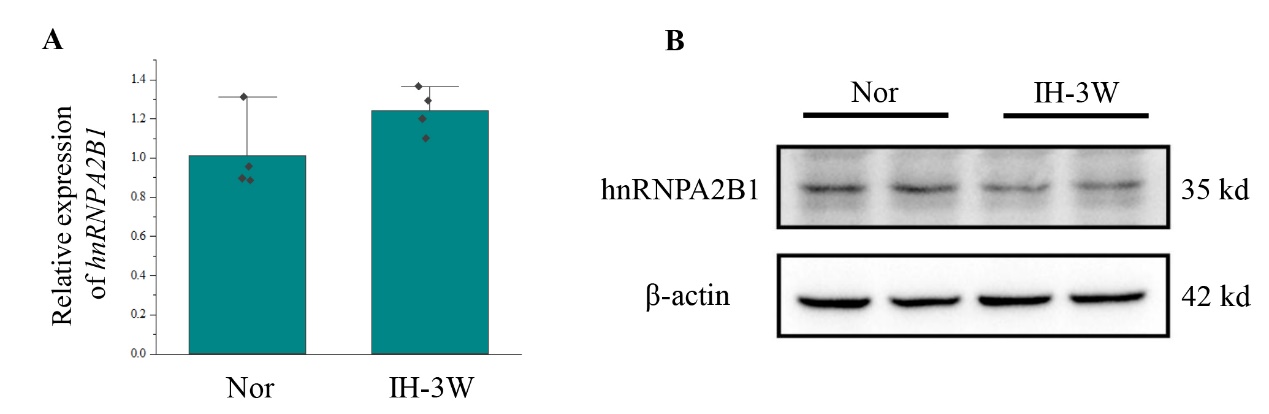
Supplementary Figure 4 Detection of hnRNPA2B1 expression in hippocampus after intermittent hypoxia**. (A) RT-qPCR was used to detect the expression of hnRNPA2B1 in the hippocampus after intermittent hypoxia for 3 weeks. The reference genes were β-actin. N=4. The Mann-Whitney U test was used for statistical analysis. (B) Western blot was used to detect the expression of hnRNPA2B1 in the hippocampus after intermittent hypoxia for 3 weeks. The reference protein was β-actin.


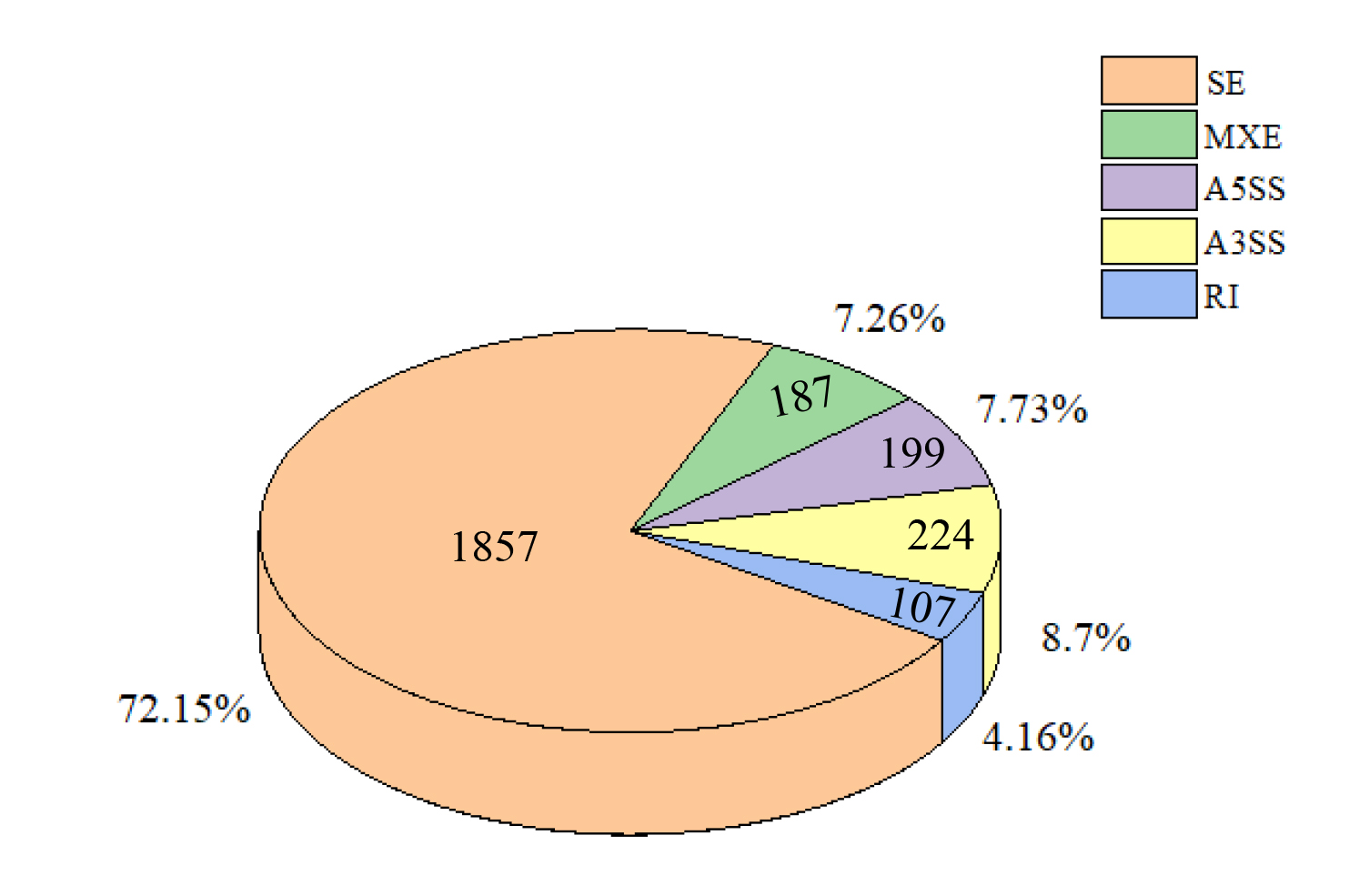


**Supplementary Figure 5 Pie chart of differential alternative splicing events.** Pie chart of differential alternative splicing events (p-adjust < 0.05) induced by intermittent hypoxia for 3 weeks. SE: skipped exon; RI: retained intron; MXE: mutually exclusive exons; A5SS: alternative 5’ splice site; A3SS: alternative 3’ splice site.

**Supplementary Figure 6 Detetion of exon inclusion of *Ep400*.** (A) Exon inclusion of *Ep400* was detected by semi-quantitative PCR. (B) Quantification of the transcripts in (A) and calculation of the ratio of exon inclusion of *Ep400*, N=4.


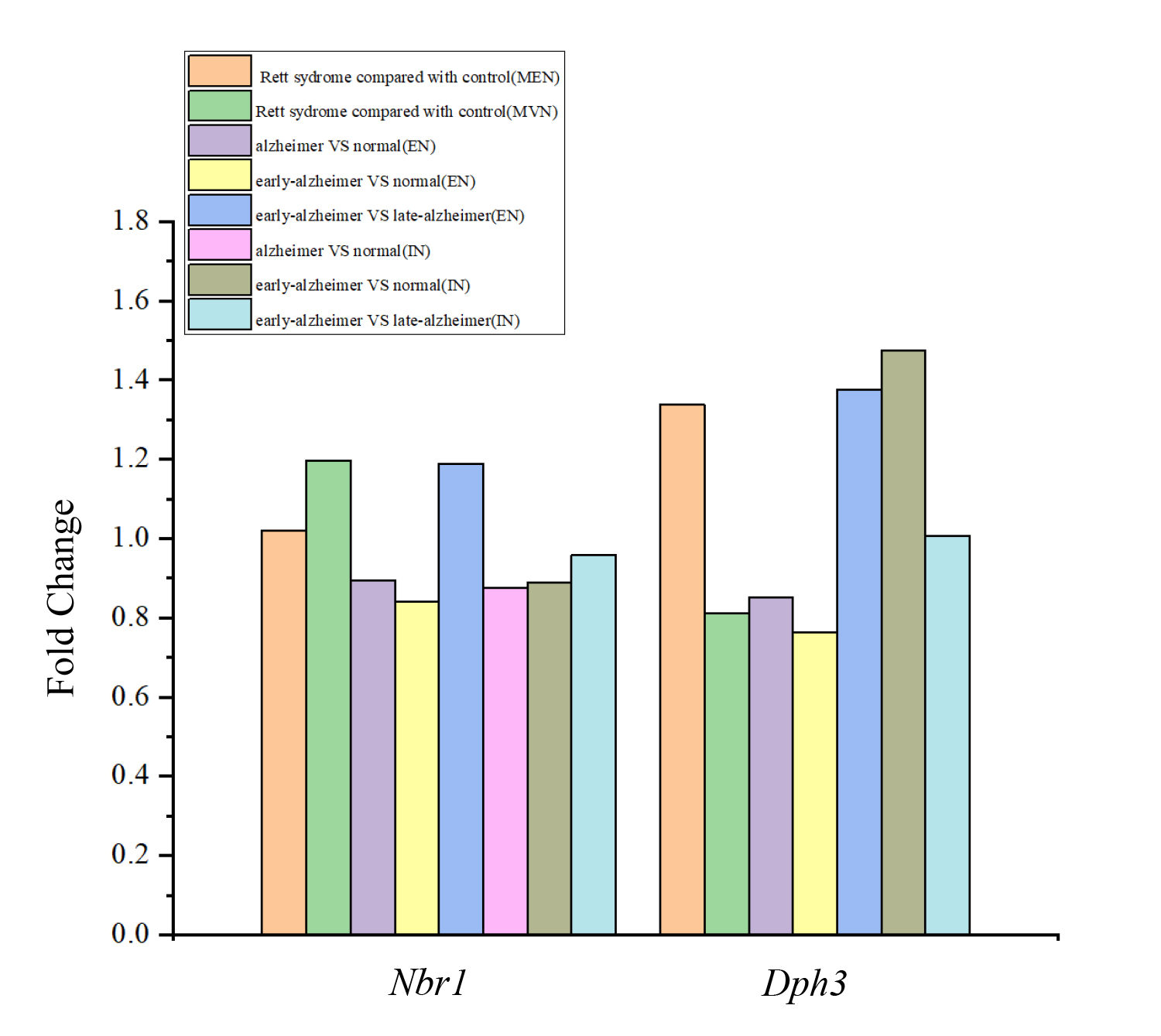

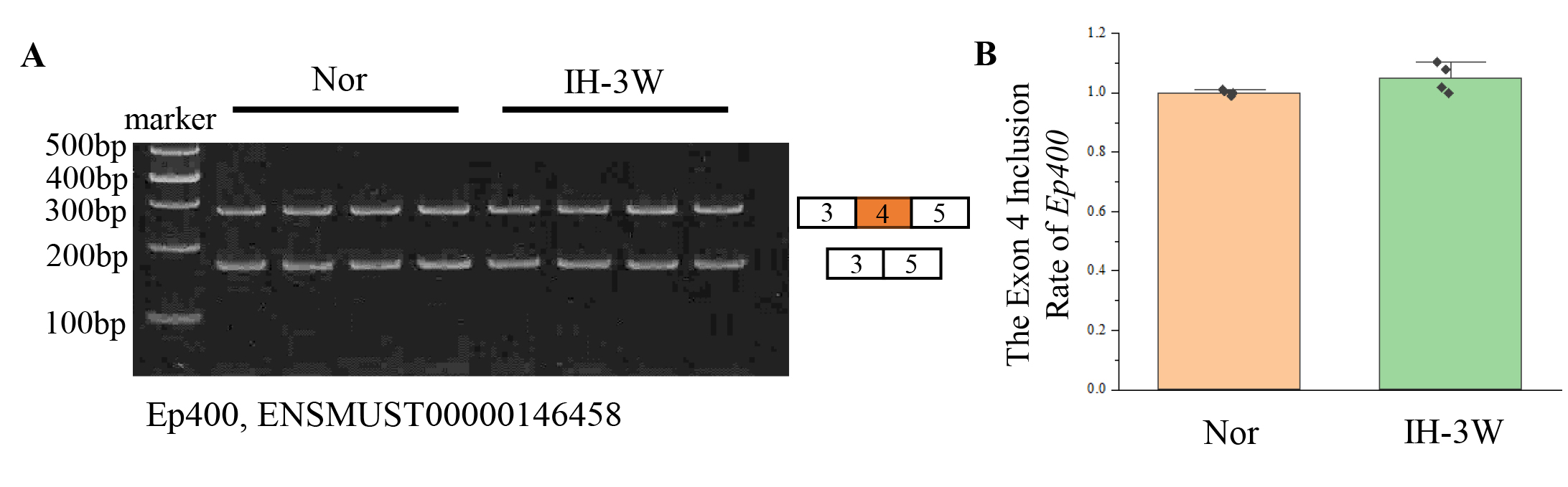


**Supplementary Figure 7 Association analysis of *Nbr1* and *Dph3* with neurological disorders.** The expression of *Nbr1* and *Dph3* in various neurological diseases was analyzed using the SC2disease database. MEN: Mutant excitatory neurons; MVI: Mutant VIP interneurons; EN: Excitatory neurons; IN: Inhibitory neurons.


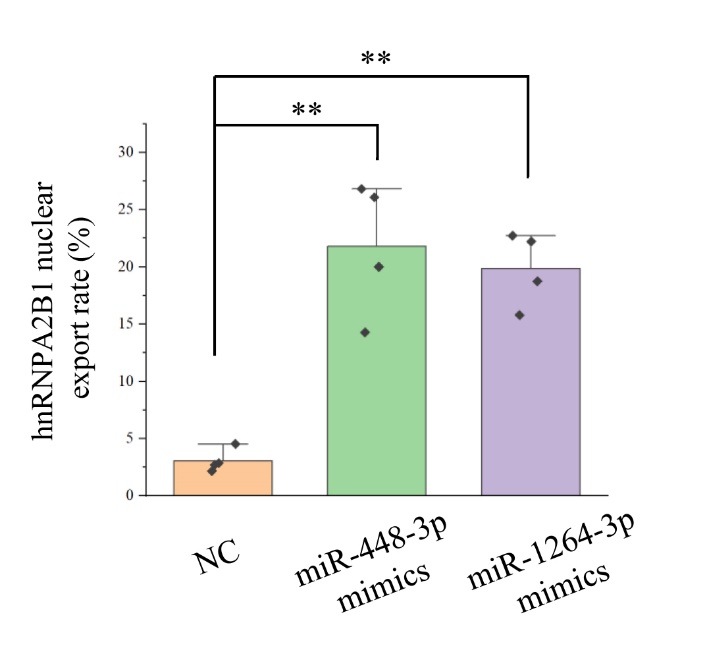


**Supplementary Figure 8 Statistics of the proportion of cells expressing hnRNPA2B1 in the cytoplasm.** After 36 h of mimics transfection, the proportion of cells expressing hnRNPA2B1 in the cytoplasm was counted (Figure 5A). N=4, **: *p* < 0.01.
